# Diseases and Causes of Death in European Bats: Dynamics in Disease Susceptibility and Infection Rates

**DOI:** 10.1371/journal.pone.0029773

**Published:** 2011-12-28

**Authors:** Kristin Mühldorfer, Stephanie Speck, Andreas Kurth, René Lesnik, Conrad Freuling, Thomas Müller, Stephanie Kramer-Schadt, Gudrun Wibbelt

**Affiliations:** 1 Leibniz Institute for Zoo and Wildlife Research, Berlin, Germany; 2 Bundeswehr Institute of Microbiology, Munich, Germany; 3 Robert Koch Institute, Berlin, Germany; 4 Friedrich-Loeffler-Institute, Wusterhausen, Germany; Veterinary Laboratories Agency, United Kingdom

## Abstract

**Background:**

Bats receive increasing attention in infectious disease studies, because of their well recognized status as reservoir species for various infectious agents. This is even more important, as bats with their capability of long distance dispersal and complex social structures are unique in the way microbes could be spread by these mammalian species. Nevertheless, infection studies in bats are predominantly limited to the identification of specific pathogens presenting a potential health threat to humans. But the impact of infectious agents on the individual host and their importance on bat mortality is largely unknown and has been neglected in most studies published to date.

**Methodology/Principal Findings:**

Between 2002 and 2009, 486 deceased bats of 19 European species (family *Vespertilionidae*) were collected in different geographic regions in Germany. Most animals represented individual cases that have been incidentally found close to roosting sites or near human habitation in urban and urban-like environments. The bat carcasses were subjected to a post-mortem examination and investigated histo-pathologically, bacteriologically and virologically. Trauma and disease represented the most important causes of death in these bats. Comparative analysis of pathological findings and microbiological results show that microbial agents indeed have an impact on bats succumbing to infectious diseases, with fatal bacterial, viral and parasitic infections found in at least 12% of the bats investigated.

**Conclusions/Significance:**

Our data demonstrate the importance of diseases and infectious agents as cause of death in European bat species. The clear seasonal and individual variations in disease prevalence and infection rates indicate that maternity colonies are more susceptible to infectious agents, underlining the possible important role of host physiology, immunity and roosting behavior as risk factors for infection of bats.

## Introduction

Bats are among the most successful and diverse mammals on earth. Approximately 1230 chiropteran species are found on every continent except Antarctica and inhabit a multitude of diverse ecological niches [1]. Bats play essential roles in maintaining healthy ecosystems, as they act as plant pollinators, seed dispersers, and predators of populations of insects including harmful forest and agricultural pests [2]. Most bat species are listed in the IUCN Red list of endangered species and almost half of these are considered threatened or near-threatened [3]. To estimate and prevent further population declines, research has been primarily focused on bat biology, ecology and behavior, while disease aspects were largely neglected [4].

In the last two decades, the importance of chiropteran species as potential vectors of significant viral diseases especially in regard to zoonoses has received growing attention. Besides bat rabies that has been studied for more than half a century, extensive research efforts identified a large number of microbial agents [5] including important emerging zoonotic viruses detected in bats across the world [6]–[12]. However, most studies are limited to the identification of microorganisms detected and investigations regarding infectious diseases and causes of death in bats are sparse [13]–[16].

In Europe, research is predominantly focused on European bat lyssaviruses [17], [18] and coronaviruses [19], [20], but first indications of bat-pathogenic bacteria [13], [14], [21]–[23] and novel viruses [24], [25] isolated from deceased bats in Germany and Great Britain were found. In this study, we provide new data on infectious diseases in European bat species, considering factors likely to affect the susceptibility of bats to infectious agents including effects of seasonality, individual and species-specific heterogeneities, and possible intra- and inter-species transmission dynamics.

## Materials and Methods

All bat species in Europe are strictly protected under the Flora-Fauna-Habitat Guidelines of the European Union (http://ec.europa.eu/environment/nature/legislation/habitatsdirective/index_en.htm) (92/43/EEC) and the Agreement on the Conservation of Populations of European Bats (www.eurobats.org) that prohibit invasive sampling of bats for research purposes. For the animals investigated in this study, carcasses of deceased bats found in Germany were kindly provided by bat researchers and bat rehabilitation centers of different federal states.

### Study material

**Figure 1:**
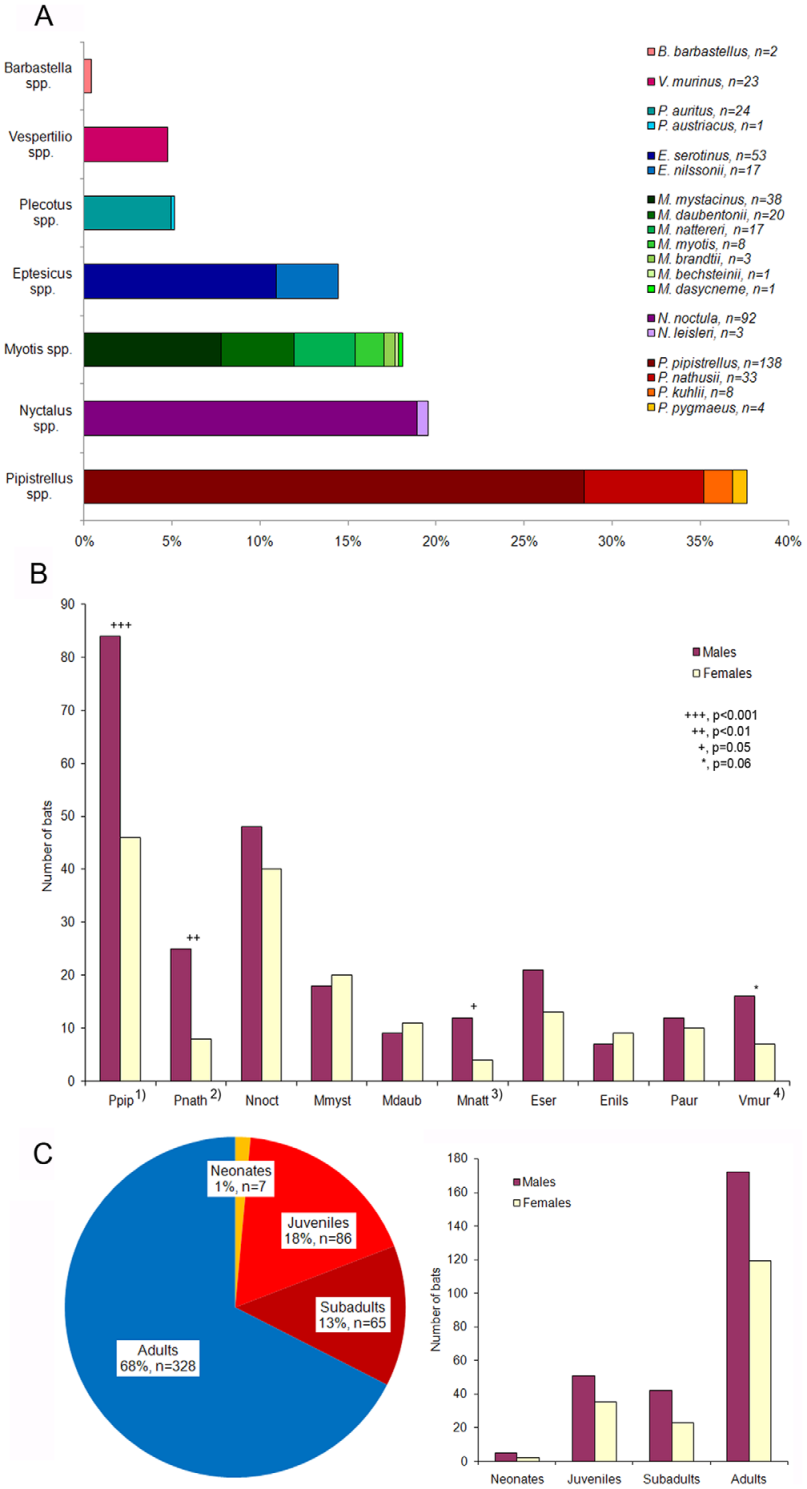
Details on bats from Germany. (A) Bat species distribution among the study sample (n = 486). (B) Male-to-female ratio (bat species >10 individuals). Footnotes: 1) Chi-square test, χ^2^ = 11.1, df = 1, p = 0.0009, 2) χ^2^ = 8.8, df = 1, p = 0.003, 3) χ^2^ = 4.0, df = 1, p = 0.05, 4) χ^2^ = 3.5, df = 1, p = 0.06. Abbreviations: Ppip, *Pipistrellus pipistrellus*; Pnath, *Pipistrellus nathusii*; Nnoc, *Nyctalus noctula*; Mmyst, *Myotis mystacinus*; Mdaub, *Myotis daubentonii*; Mnatt, *Myotis nattereri*; Eser, *Eptesicus serotinus*; Enils, *Eptesicus nilssonii*; Paur, *Plecotus auritus*; Vmur, *Vespertilio murinus*. (C) Age-sex distribution among the study sample (n = 486).

Between 2002 and 2009, a total of 486 deceased bats of 19 European vespertilionid species (i.e., Family V*espertilionidae*) were investigated (Fig. 1A, [26]). The bat carcasses originated from 6 different geographic regions in Germany, i.e. Berlin greater metropolitan area (n = 223), Bavaria (n = 165), Brandenburg (n = 38), Lower Saxony (n = 36), Thuringia (n = 21), and Baden-Wuerttemberg (n = 3), and were collected by bat researchers and bat rehabilitation centers. Most animals represented individual cases that were found dead, injured or moribund near human habitation. Thus, the species composition in this study predominately reflected the urban and suburban bat fauna, which is characterized by a disproportionate abundance of a few bat species (Fig. 1A, [27], [28]). Two groups of 2 and 21 adult noctules (*Nyctalus noctula*), respectively, were collected from tree hibernacula destroyed during wood logging. A further group of 25 deceased adult *N. noctula* originated from a colony that was trapped in a rain pipe in December. Nine dead juvenile *Pipistrellus pipistrellus* were collected from a nursery roost.

If bats died in care or had to be euthanized for animal welfare reasons, the carcasses were immediately stored at −20°C and were shipped to the Leibniz Institute for Zoo and Wildlife Research, Berlin, Germany, for diagnostic investigations. Of all carcasses examined histo-pathologically, about 90% were suitable for bacteriological investigation. A lesser extend (43%) was also examined for selected viral agents at the Robert Koch Institute, Berlin, Germany. In addition, a brain sample of each animal was submitted to the Friedrich-Loeffler-Institute, Wusterhausen, Germany, for rabies diagnosis.

### Pathological investigation

A full necropsy was performed on each bat and all macroscopic findings including ectoparasite infestation were recorded. For histo-pathological examination, small slices of multiple organ tissues (i.e., lung, liver, heart, kidney, adrenal gland, spleen, intestine, pancreas, brain, tongue, larynx, salivary gland and pectoral muscle) and tissues conspicuous for pathological changes were fixed in buffered 4% formalin, processed using standard methods and embedded in liquid paraffin. Sections were cut at 2–5 µm and routinely stained with hematoxylin-eosin (HE). In addition, special histological staining methods were used depending on microscopic findings, i.e. for the detection of bacteria (Gram or Giemsa staining), fungi (periodic acid Schiff or Grocott's Gomori methenamine silver nitrate staining), iron (Prussian blue stain), mineralization (von Kossa staining), connective and collagen tissue (trichrome staining). Details on pathological results are published elsewhere [26].

The causes of mortality were rigorously standardized with the primary cause of death identified for each bat as the most serious injury, disease or event subsequently fatal to the animal. To ensure independence of primary and contributing causes of death, the categorization was based on the severity of pathological findings.

### Bacteriological investigation

Samples of lung, liver, heart and kidney, and tissues conspicuous for pathological changes (e.g. enlarged spleen) of 430 bats were plated onto Columbia (5% sheep blood), Chocolate, Gassner, and MacConkey agar (Oxoid, Germany) and were incubated at 37°C (Chocolate agar 5% CO_2_) for 24–48 h. Specific culture media and conditions for the isolation of *Yersinia*, *Salmonella* and anaerobic bacteria were used if appropriate. Primary identification of bacterial strains was based on colony morphology, hemolysis, Gram-staining, indol production, catalase and oxidase reaction. Bacterial species identification was carried out using the relevant commercial Api test system (bioMérieux, Germany). Additional conventional biochemical tests [29], [30] were applied to confirm Api test results where necessary. In case of ambiguous biochemical test results, 16S rDNA gene analysis was performed for final identification [23]. *Salmonella* isolates were characterized at the National Reference Laboratory for the Analysis and Testing of Zoonoses (*Salmonella*) at the Federal Institute for Risk Assessment, Berlin, Germany. Identification and characterization of *Yersinia* and *Pasteurella* species have been reported earlier [22], [23].

### Virological investigation

Homogenized organ tissue of lung, liver, heart, kidney, spleen, brain and salivary gland of 210 bats were pooled for each individual and used for RNA/DNA extraction and further molecular analysis by generic PCR assays detecting flavi- [31], hanta- [32], corona- [33], and influenza A-viruses [34]. Also, PCR assays specific for 8 previously described herpesviruses [24] from European vespertilionid bats were used. For this purpose, RNA/DNA was isolated using the NucleoSpin® RNA II Kit (Macherey-Nagel, Germany) and the NucleoSpin® Tissue Kit (Macherey-Nagel), respectively, according to the manufacturer's instructions. Because of limitations in sample volume, for 180 out of the 210 bats PCR assays could only be applied for 4 different bat herpesviruses. Internal controls were used for all PCR assays to test for inhibition. For confirmation, all retrieved fragments of bat herpesvirus-specific PCR assays were checked for sequence identity to previously published isolates [24].

For detection of lyssavirus antigen in brain tissue the fluorescent antibody test (FAT) using a polyclonal antirabies conjugate (Sifin, Germany) was used [35]. FAT-positive brain tissues were subject of virus isolation in murine neuroblastoma cell culture (Na 42/13) using the Rabies Tissue Culture Infection Test (RTCIT) as described elsewhere [36]. Lyssaviruses isolated in cell culture were characterized using both a panel of 10 anti-nucleocapsid monoclonal antibodies (MAb) [37] and partial sequencing of a fragment of the nucleoprotein gene after RNA extraction using Trizol (Invitrogen, Germany) essentially as described [18].

### Genetic identification of bat species

Genomic DNA was extracted from organ homogenates using the NucleoSpin® Tissue Kit (Macherey-Nagel) according to manufacturer's recommendations. Genetic identification of the bat species was performed by amplification and sequencing of a 241 bp fragment of the cytochrome B (*cytB*) gene [38] using primers FM up (5′- CCC CHC CHC AYA TYA ARC CMG ART GAT A -3′) and FM down (5′- TCR ACD GGN TGY CCT CCD ATT CAT GTT A -3′). In addition, for differentiation of the 2 distinct *Pipistrellus* species, *P. pipistrellus* and *P. pygmaeus*, a rapid multiplex PCR assay was performed as described by Kaňuch et al. [39] using primers PIP-F (5′- CTC ATT CAT TGA YCT ACC AGC -3′), PIP-R (5′- CAG CRA ATA GTA AAA TAA CTC C -3′) and Ppip-F (5′- CAT CTG TTT GGG ACT ACA GAT CC -3′).

### Statistical analysis

The bat data were categorized in regard to different explanatory numeric and factor variables, e.g. bat species, sex and age class. The variable ‘age class’ ranked between 1 and 4 with increasing age (i.e. neonates, juveniles, subadults, and adults) and was used as numeric variable. For endoparasitic analysis, we defined a 3 level variable ‘bat size’ according to the body size of a certain bat species to reduce the degrees of freedom of the full model, i.e. large species (*N. noctula*, *Eptesicus serotinus*, and *Vespertilio murinus*), medium-sized species (*E. nilssonii*, *Plecotus auritus*, *Myotis daubentonii*, *M. nattereri*, and *P. nathusii*) and small species (*P. pipistrellus*, and *M. mystacinus*). To detect effects of seasonality, 4 different activity periods were specified according to the date of sampling, i.e. hibernation period (November to March), post-hibernation period (April/May), maternity period (June to August), and swarming period (September/October). As dependent binary variable for the respective models we either classified the mortality cause being disease or not (i.e. trauma), or the presence-absence of bacterial, ecto- and endoparasitic infections.

We formulated 4 different hypotheses to test for individual and species-specific differences in disease susceptibility and infection rates: (A) Disease-related mortality in bats is influenced by sex, age and species-specific differences, and degree of endoparasitic infection. (B) Bacterial infection in bats is influenced by sex, age and species-specific differences, occurrence of traumatic injuries and cat predation. (C) Ecto- or (D) endoparasitic infection in bats is affected by age, sex and species-specific differences. Seasonal effects were not analyzed because of too many missing data points. Because the long-term dataset was highly biased towards sampling procedure, preservation of bat carcasses and following diagnostic investigations, we split and filtered the full data into several subsets reflecting the different analyses (Table 1).

**Table 1:** Description of the data sets used for different analyses.

	Analysis	Data set(total n)	Sex(% males)	Age(% adults)	Bat species(total n)
Full dataset	Bat samples	486	55.6	67.5	19
Subset 1	Causes of death	433^a^	55.0	65.4	19
	GLMM: disease- vs. trauma-related mortality (A)	289^a^	55.0	65.7	17
Subset 2	Bacteriological results	430	58.4	65.3	18
	GLMM: bacterial infection vs. no infection (B)	377^a^	58.1	62.6	18
Subset 3	Virological results	210^b^	56.7	64.3	16
Subset 4	Parasitological results	433^a^	55.0	65.4	19
	GLM: parasitic infection vs. no infection (C, D)	402^a^	54.7	65.2	10

GLMM, generalized linear mixed models with bat species included as random effect.

GLM, generalized linear models for datasets with bat species >10 individuals.

A–D: refers to the models analyzed on the different data sets (see chapter ‘Statistical analyses’).

a To avoid overrepresentation of bat samples that were collected at the same time and location, a randomly selected individual of each group was included in the final dataset.

b For detection of lyssavirus antigen, brain tissue of all 486 bats was tested.

All statistical analyses were performed using the R software V. 2.13.1 (R Development Core Team 2011, Vienna, Austria). We used the chi-square test for given probabilities to evaluate significant differences in the sex ratio among bats of different species. For hypotheses A and B, we used a generalized linear mixed modeling approach (binomial GLMM using function lmer in library lme4) with bat species included as random effect. This variable had not been significant as fixed effect (results not shown), but from other studies we can assume that there are species-specific differences in susceptibility of bats to certain infectious agents and therefore included it as random effect. We further used generalized linear models (GLM with logit link and binomial error structure; for datasets with bat species >10 individuals) to test for individual and species-specific differences in parasite infection rates (hypotheses C and D).

We created a full model for each hypothesis (A–D) to examine multiple and interaction effects of the specified variables. To select the final model variables, we used a stepwise backward algorithm (function stepAIC in library MASS) based on Akaike's information criterion (AIC) [40]. The ΔAIC of the final model was calculated relative to a random intercept model to demonstrate the effect size of the selected variables.

## Results

Results of the diagnostic analyses follow the full data splitting into several subsets (see section ‘Statistical analysis’ in Material and Methods; Table 1).

### *Full dataset:* Bat samples

All sampled bats belonged to 7 different genera (i.e. *Pipistrellus*, *Nyctalus*, *Myotis*, *Eptesicus*, *Plecotus*, *Vespertilio*, and *Barbastella*) and 19 European vespertilionid species (Fig. 1A). Three bat species, the common pipistrelle (*P. pipistrellus*, n = 138), the noctule bat (*N. noctula*, n = 92), and the serotine bat (*E. serotinus*, n = 53) constituted about 60% of all bat carcasses investigated in this study, whereas *P. pygmaeus*, *Nyctalus leisleri*, *Myotis brandtii*, *M. bechsteinii*, *M. dasycneme*, *Plecotus austriacus* and *Barbastella barbastellus* were represented in small numbers of 1 to 4 animals. The overall sex ratio was 1.5 males to 1 female with significant species-specific differences (Fig. 1B). Animals in their first year of life (neonates, juveniles, and subadults) represented one third (32.5%, n = 158) of bat samples (Fig. 1C).

### *Subset 1:* Causes of death

Overall, we were able to assign a cause of death to 70% (n = 304) of bats investigated in this study. Two thirds of mortality were due to trauma (n = 145) or disease (n = 144), while almost 4% of bats had died of other non-infectious causes like pulmonary edema, dehydration and hypoglycemia (Table 2). In 30% (n = 129) no significant pathological findings could be found.

**Table 2:** Causes of mortality of bats from Germany.

				Age class		Sex class		
Cause of death	n	%	Euthanasia	<1 Year	Adult	Female	Male	n.d.
**Trauma**	145	33.5	54	41	104	55	87	3
Unknown trauma cause	71	16.5	29	19	52	33	36	3
Cat predation	66	15.3	23	19	47	18	47	-
Roost destruction^a^	2	0.5	-	-	2	2	-	-
Trapped in rain pipe^a^	1	0.2	-	-	1	-	1	-
Trapped in window	1	0.2	-	-	1	-	1	-
Trapped in lamp	1	0.2	-	1	-	-	1	-
Trapped in fly strip	1	0.2	-	1	-	1	-	-
Barbed wire injury	1	0.2	1	1	-	1	-	-
Smoke poisoning	1	0.2	1	-	1	-	1	-
**Disease**	144	33.3	7	58	86	64	72	8
Unknown etiology	81	18.7	3	35	46	35	38	8
Bacterial infection	54	12.5	2	20	34	27	27	-
Viral infection^b^	5	1.2	1	1	4	1	4	-
Parasitic infection	2	0.5	-	-	2	-	2	-
Aspiration pneumonia	1	0.2	-	1	-	1	-	-
Bone deformation	1	0.2	1	1	-	-	1	-
**Others**	15	3.4	-	6	9	6	9	-
Pulmonary edema	9	2.1	-	3	6	1	8	-
Dehydration	2	0.5	-	-	2	1	1	-
Anemia^c^	1	0.2	-	-	1	1	-	-
Hyperthermia^d^	1	0.2	-	1	-	1	-	-
Hypothermia	1	0.2	-	1	-	1	-	-
Hypoglycemia	1	0.2	-	1	-	1	-	-
**No significant findings**	129	29.8	1	45	84	33	70	26
**Total**	433	100	62	150	283	158	238	37

n.d., not determined.

a A randomly selected individual of 3 different groups of adult *Nyctalus noctula*.

b Adenovirus (bat AdV-2) [25] and European bat lyssavirus (EBLV-1) infection.

c Due to severe tick infestation.

d A randomly selected individual of a group of juvenile *Pipistrellus pipistrellus*.

Among the 145 traumatized bats, additional mild (n = 42), moderate (n = 28) and severe (n = 4) inflammatory organ changes were noted in one half (50.9%) of individuals, and 23% of the bats revealed bacterial (n = 19) and/or parasitic infections (n = 15) (Table 3). Of the 144 bats considered as dying of disease, fatal bacterial (n = 54), viral (n = 5) and parasitic infections (n = 2) were observed in 42%. Besides, amniotic fluid aspiration was noted in a neonate noctule bat (*N. noctula*), and a juvenile common pipistrelle (*P. pipistrellus*) was euthanized because of severe forearm bone deformation. The remaining 81 bats (56.3%) revealed moderate to severe pathological changes of unknown etiology or unconfirmed bacterial or viral cause (Table 2).

**Table 3:** Pathological findings and bacterial, viral and parasitic infections specified for the general causes of mortality, trauma and disease.

	Trauma		Disease	
	n	%	n	%
Total number of bats	145	33.5	144	33.3
**Pathological findings** a				
Injuries	136	93.8	37	25.7
Inflammatory lesions	74	51.0	124	86.1
Non-inflammatory lesions	1	0.7	20	13.9
Spleen activation	81	55.9	82	56.9
Circulatory changes	53	36.3	41	28.5
Metabolic disorders	10	6.8	12	8.3
**Bacterial infection**	19	13.0	54	37.5
**Viral infection** b	-	-	5	3.5
**Parasitic infection** c	15	10.3	14	9.7

a Details on pathological findings described elsewhere [26].

b Adenovirus (bat AdV-2) [25] and European bat lyssavirus (EBLV-1) infection.

c Severe intestinal trematode infection, disseminated nematode infection, renal or intestinal coccidiosis [26].

**Figure 2:**
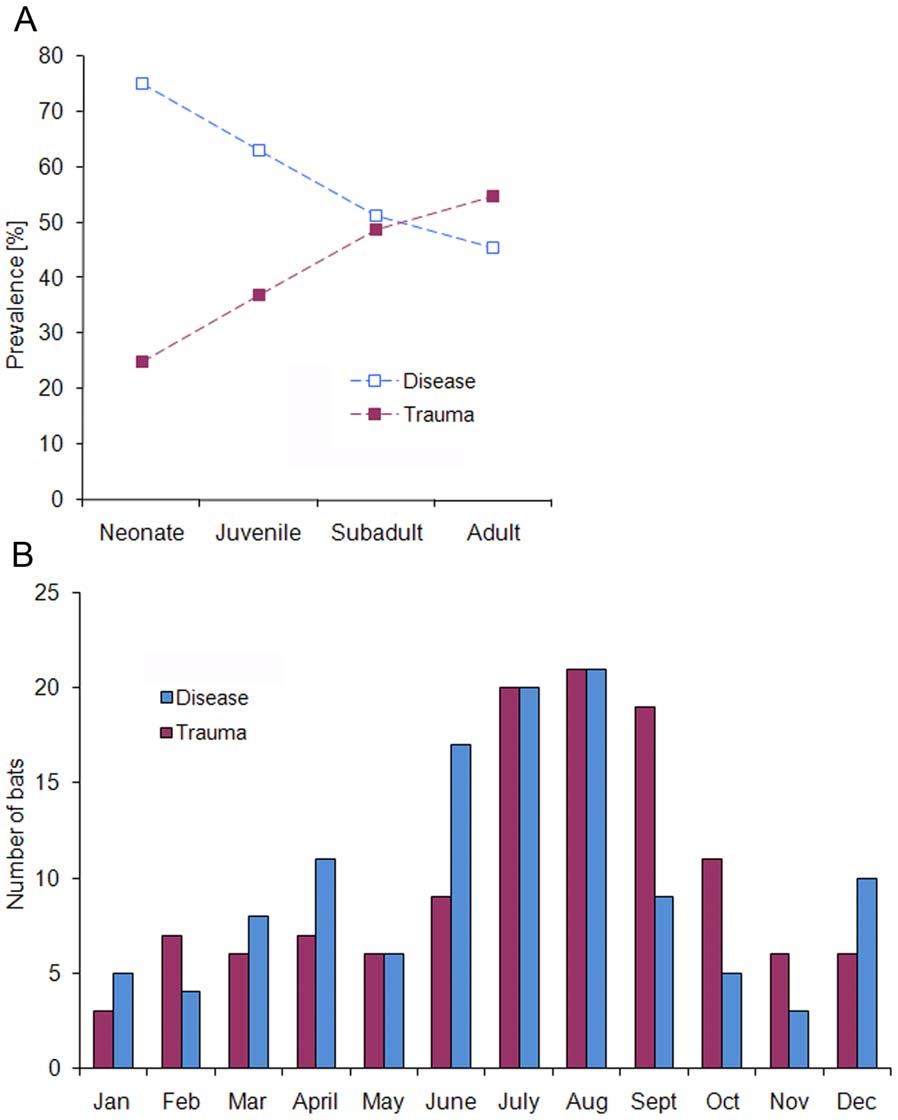
Age-dependent differences and seasonal variations among the general causes of mortality, disease and trauma. (A) Age-specific prevalence. (B) Seasonal distribution of trauma- and disease-related mortality cases.

Based on the GLMM analysis, significant age- and sex-dependent differences (ΔAIC = 23.13) were detected between the general causes of mortality, disease and trauma (Table 4). The disease presence in bat samples decreased continuously with increasing age. Neonates and juveniles of both sexes were significantly more affected by disease than older age classes (Table 4; Fig. 2A). We also found a significant trend in disease-associated mortality between the sexes, with adult females displaying higher disease prevalence (52.5%) than males (36.4%) (Table 4). No significant association was observed between a certain cause of mortality (i.e. disease or trauma) and severity of endoparasitic infection (ΔAIC = 0.75, result not shown). The seasonal distribution of disease-related mortality cases (Fig. 2B) described a trimodal pattern, with peaks in spring (April), summer (June to August) and winter (December). The proportion of traumatized individuals also increased obviously during the summer months up to and including the swarming period, but was low during the rest of the year.

**Table 4:** Result of the final model variables corresponding to 4 different analyses: (A) disease- vs. trauma-related mortality, and presence-absence of (B) bacterial, (C) ecto- and (D) endoparasitic infection.

	Analysis	ΔAIC*	Variable	Factor level	Estimate	SE	z-value	p-value
(A)	GLMM	23.13	Age class		−0.56	0.18	−3.09	0.002
			Sex (male)		−0.62	0.28	−2.19	0.03
(B)	GLMM	16.00	Cat predation		1.20	0.28	4.32	<0.0001
(C)	GLM	14.58	Bat species	*Nyctalus noctula*	−0.30	0.30	−1.02	0.3
				*Myotis daubentonii*	−1.10	0.52	−2.13	0.03
				*Vespertilio murinus*	−1.56	0.55	−2.83	0.005
				*Eptesicus nilssonii*	−2.01	0.75	−2.68	0.007
				*Pipistrellus pipistrellus*	−2.04	0.27	−7.42	<0.0001
				*Eptesicus serotinus*	−2.06	0.43	−4.75	<0.0001
				*Plecotus auritus*	−2.40	0.74	−3.25	0.001
				*Pipistrellus nathusii*	−2.74	0.73	−3.76	0.0002
				*Myotis nattereri*	−2.77	1.03	−2.69	0.007
				*Myotis mystacinus*	−2.90	0.73	−3.98	<0.0001
(D)	GLM	24.95	Age class		0.43	0.15	2.88	0.004
			Bat size	Large species	−0.18	0.18	−0.99	0.3
				Medium-sized species	−1.30	0.23	−5.64	<0.0001
				Small species	−1.29	0.19	−6.86	<0.0001

GLMM, generalized linear mixed models with bat species included as random effect.

GLM, generalized linear models for datasets with bat species >10 individuals.

AIC, Akaike's information criterion.

*ΔAIC of the final model relative to a random intercept model.

### *Subset 2:* Bacteriological results

About 90% (n = 430) of bat samples were examined bacteriologically. Among these, 42 different bacterial genera with more than 53 bacterial species were identified (Table S1). Predominant bacteria isolated were *Enterococcus faecalis* (14.7%, n = 63), *Hafnia alvei* (11.2%, n = 48), *Serratia liquefaciens* (10%, n = 43), and *Pasteurella multocida* (7.7%, n = 29). In 37% (n = 157) of bats no bacterial growth was observed at all.

Comparative bacteriologic and histo-pathologic analysis identified 22 different bacterial species that were clearly associated with pathological lesions and/or systemic infection, found in 17% (n = 73) of bats investigated bacteriologically (Table 5). Members of the families *Pasteurellaceae* (above all *P. multocida*) (41.1%, n = 30), *Enterobacteriaceae* (various bacterial species) (28.8%, n = 21), and *Streptococcaceae* (above all *Enterococcus* spp.) (21.9%, n = 16) were predominant bacteria associated with disease. More than half (54.8%, n = 40) of bacterial infections were observed in bats with traumatic injuries. The GLMM analysis revealed low sex- and age-dependent differences in bacterial infection (ΔAIC = 1.97, result not shown). Female bats (21.9%) and adults (21.6%) showed marginally higher prevalence of bacterial disease compared to males (18.3%) and to other age classes (15.6%), respectively. However, we found a strong influence of cat predation (ΔAIC = 16) associated with bacterial infection in bats (Table 4).

**Table 5:** Bacteria associated with disease in bats from Germany.

Bacteria	Bats	Clinical status*
*Pasteurella multocida*	28	Septicemia; pneumonia; pleuritis; peri-/epicarditis; myocarditis; nephritis; liver/spleen necroses; wound infection; abscess
*Pasteurella multocida, Pasteurella* species B	1	Septicemia; glossitis (bite wound infection); liver necrosis
*Pasteurella pneumotropica, Vibrio* spp.	1	Septicemia
*Serratia liquefaciens*	5	Systemic infection; pneumonia; wound infection
*Serratia marcescens*	1	Systemic infection; pneumonia
*Enterobacter cancerogenus*	2	Systemic infection; pneumonia
*Enterobacter cancerogenus, Hafnia alvei*	1	Peritonitis; pneumonia
*Hafnia alvei*	1	Systemic infection
*Klebsiella oxytoca*	3	Systemic infection; pneumonia
*Klebsiella mobilis*	1	Systemic infection; pneumonia
*Escherichia coli*	2	Systemic infection; pneumonia; nephritis; cystitis
*Salmonella enterica* serotype Typhimurium	2	Systemic infection; pneumonia; meningitis
*Salmonella enterica* serotype Enteritidis	1	Systemic infection; pneumonia, wound infection
*Yersinia pseudotuberculosis*	1	Systemic infection; pneumonia; liver/spleen necroses
*Cedecea davisae*	1	Pneumonia
*Burkholderia* sp.	1	Systemic infection
*Enterococcus faecalis*	9	Septicemia; pneumonia; endocarditis; abscess
*Enterococcus faecium*	3	Septicemia; pneumonia
*Enterococcus faecalis, Enterococcus faecium*	2	Septicemia; pneumonia; myocarditis; wound infection
*Staphylococcus aureus*	3	Septicemia
*Staphylococcus aureus, Enterococcus faecalis*	1	Septicemia; dermatitis
*Aerococcus viridans*	1	Systemic infection; pneumonia
*Bacillus* sp.	1	Pneumonia
*Clostridium sordellii*	1	Hemorrhagic enteritis

*Histo-pathological findings described in more details elsewhere [26].

### *Subset 3:* Virological results

Testing for human-pathogenic zoonotic viruses, no examined bat sample (0/210) was positive for influenza A virus, corona-, hanta- and flaviviruses, respectively. No inhibition of the PCR assays was notified. Out of 486 bats tested for rabies virus infection, 2 serotine bats (*E. serotinus*) were positive for lyssavirus by FAT and RTCIT. The viruses were identified as European bat lyssavirus type 1 (EBLV-1) sublineage a, both using MAbs and sequencing.

Applying bat herpesvirus-specific PCR assays, 63 out of 210 bats proved to be infected with 7 of the previously described 8 bat herpesviruses (Table 6). The highest prevalence of 65% (24/37) was observed for bat gammaherpesvirus 6 (BatGHV6) in common pipistrelle bats (*P. pipistrellus*), followed by bat gammaherpesvirus 5 (BatGHV5, 42.1%) in nathusius' pipistrelle bats (*P. nathusii*) and bat gammaherpesvirus 4 (BatGHV4, 33.8%) in noctule bats (*N. noctula*). Co-infection with different bat herpesviruses were recognized in 4 *N. noctula* (7.4%) infected with BatGHV3 and BatGHV4, and in one *N. noctula* (1.5%) infected with BatGHV4 and BatGHV5. BatGHV5 was not only detected in its initially specific host *P. nathusii*, but also in 3 other bat species, i.e. *N. noctula*, *Myotis myotis* and *M. mystacinus*. Although the prevalence of BatGHV3 (13.0%) and BatGHV4 (33.8%) differed significantly within its migrating host *N. noctula*, no difference was observed between the sexes. Two juvenile *N. noctula* were found to be infected with BatGHV4. Interestingly, for the sedentary bat species *P. pipistrellus* being infected with BatGHV6, a considerably higher prevalence was observed in 22 juvenile bats (72.7%) resulting in an overall prevalence of 65% also without difference between adult male and female bats.

**Table 6:** Bat herpesvirus infection in bats from Germany.

Virus	Bat species	Total	Positive (%)	
Bat herpesviruses	16 species	210	63	(30.0)
BatGHV1^a^	*Eptesicus serotinus*	9	1	(11.1)
BatGHV3^a^	*Nyctalus noctula* c	54	7	(13.0)
BatGHV4^b^	*Nyctalus noctula* c	65	22	(33.8)
BatGHV5^b^	*Pipistrellus nathusii*	19	8	(42.1)
	*Nyctalus noctula* c	65	1	(1.5)
	*Myotis myotis*	2	1	n.d.
	*Myotis mystacinus*	21	1	(4.8)
BatGHV6^a^	*Pipistrellus pipistrellus*	37	24	(64.9)
BatGHV7^b^	*Plecotus auritus*	12	2	(16.7)
BatBHV1^b^	*Myotis nattereri*	2	1	n.d.

BatGHV, Bat gammaherpesvirus.

BatBHV, Bat betaherpesvirus.

a Tested bats from a sample set containing 180 animals.

b Tested bats from a sample set containing 210 animals.

c Co-infection of different herpesviruses recognized.

n.d., not determined due to insufficient sample numbers.

### *Subset 4:* Parasitological results

**Figure 3:**
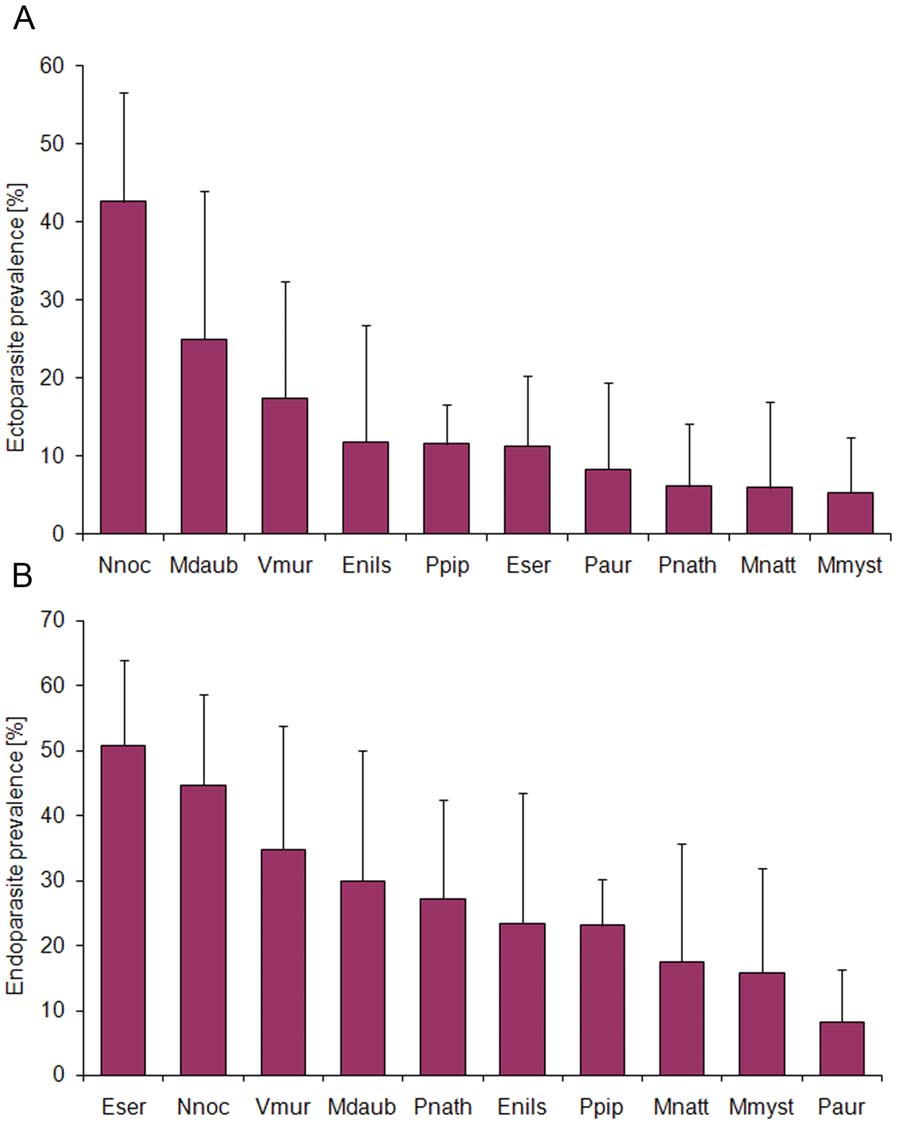
Species-specific parasite infection rates. (A) Ecto- and (B) endoparasite prevalence in different European vespertilionid bat species. Error bars represent 95% binomial confidence intervals. Abbreviations: Nnoc, *Nyctalus noctula*; Mdaub, *Myotis daubentonii*; Vmur, *Vespertilio murinus*; Enils, *Eptesicus nilssonii*; Ppip, *Pipistrellus pipistrellus*; Eser, *Eptesicus serotinus*; Paur, *Plecotus auritus*; Pnath, *Pipistrellus nathusii*; Mnatt, *Myotis nattereri*; Mmyst, *Myotis mystacinus*.

Ectoparasites (mites, fleas, and ticks) were noted in 14% (n = 62) of bats, but a potential bias in ectoparasite numbers collected from dead animals in comparison to ectoparasite abundance on live animals has to be taken in account. Female bats (17.1%) were slightly more infested by ectoparasites than males (14.7%), whereas in different age classes ectoparasite prevalence was almost balanced. The GLM analysis revealed significant species-specific differences in ectoparasite infestation (ΔAIC = 14.58, Table 4). Most bat species revealed low ectoparasite prevalence (range 5.3–11.8%), while almost 43% (n = 20) of *N. noctula* were infested with mites and/or fleas (Fig. 3A).

Microscopic examination of organ tissues revealed endoparasitic infection in 29% (n = 124) of investigated bats, involving different protozoan (families *Eimeriidae* and *Sarcocystidae*) and helminth parasites (trematodes, cestodes, and nematodes). Helminthes were predominantly found in the gastro-intestinal tract of the bats, while in some animals, granulomatous organ lesions were associated with larval migration of nematode species. Based on the GLM analysis, clear age- and species-specific differences (ΔAIC = 24.95) were observed between infected and non-infected bats (Table 4). The prevalence of endoparasitic infection in bat samples increased significantly with increasing age, whereas the increase in prevalence was more rapid between juveniles and subadults (8.5%) compared to the older age classes (4.5%). Marginal differences were also observed between the sexes, with female bats showing slightly higher (30.4%) endoparasite prevalence than males (24.4%). Regarding species-specific differences, large bats like *N. noctula*, *E. serotinus* and *V. murinus* revealed higher endoparasite prevalence compared to individuals of medium-sized or small vespertilionid species (Table 4; Fig. 3B).

## Discussion

### Causes of death and disease dynamics in bats

This study was based on a passive surveillance sampling strategy that inherently influences the composition of bats sampled for diagnostic investigations [27] and might also effect the data presented on causes of death by ecological and anthropogenic impacts of urban landscapes [41]. Trauma and disease represented the most important causes of mortality in deceased bats from Germany, which differ from results of previous studies [13]–[15] where disease-related mortality often played a subordinate role. Young bats and adult females were significantly more affected by disease, indicating that sex- and age-related disease prevalence in bats are strongly correlated with the maternal season. This assumption is further supported by the distinct increase of disease-related mortality from June to August, which corresponds to the maternity period of Central European bat species. Similar seasonal prevalence patterns in bats have also been described for parasitic (e.g. [42]–[45]) and viral infections (e.g. [19], [46], [47]). In contrast, the increase of trauma-associated mortality cases from July to October resembles 4 major behavioral activity patterns of European bat species (i.e. weaning, mating, pre-hibernal fat storage, and migration) [48] and could therefore predispose bats to trauma. However, both seasonal peaks also coincide with time and locations where sick, injured or dead bats are more likely to be discovered as well as with the seasonal roosting behavior of bats adapted on urban habitats [27]. The additional seasonal peaks of disease-associated mortality corresponded to the post-hibernal and the early hibernal period of temperate zone bats. Currently, there is a lack of knowledge of bat immunology. It is known for other mammalian species that hibernation reduces the innate and adaptive immune response; likewise an increasing risk of infection could be assumed for hibernating bats [49]. With the start of the hibernation season, large aggregations of bats originating from various colonies might enhance the risk of spreading infectious agents similar to maternity colonies. Equally, the post-hibernal increase of disease-related mortality is suggestive for reduced immunity in association with prolonged fasting during hibernation.

### Bacterial diseases and cat predation

Bacterial diseases have rarely been documented in bats. *Pasteurella* spp., here identified in 7% of bats, were the predominant bacterial pathogens reported in European bats and infection appears to be strongly correlated with cat predation [13], [14], [23], [26]. In our study, bacterial infections were confirmed in 17% of bats investigated bacteriologically. Most of these bacterial isolates represented opportunistic pathogens that usually do not harm the host unless the immune system is weakened [50] or preceding injury to natural host barriers (e.g. skin abrasion). Primary bacterial pathogens like *Samonella enterica* serovar Typhimurium, *S.* Enteritidis and *Yersinia pseudotuberculosis* [22] were identified in almost 12% of affected bats. Some of the bacterial species (e.g. *Burkholderia* sp., *Cedecea davisae* and *Clostridium sordellii*) are newly described in bats. Nevertheless, bacteriologic analyses can markedly be influenced by post-mortem bacterial invaders, freezing and storage of bat carcasses and the inability to detect certain bacteria by routine culture methods, resulting in some bacterial species that might have escaped detection.

We found a strong association between cat predation and bacterial infection in bats as almost one half of bats (44%) caught by cats were affected by bacterial disease. Various bacteria can be transmitted via cat bites [51]; hence bats attacked by cats are likely to succumb to bacterial infection even if non-fatal injuries were present. This relation has been proven for *P. multocida* infections in European bat species [13], [14], [23], [26]. On the other hand, bats already debilitated by disease might easier fall prey to predators like cats. Consequently, bats may also act as vectors for zoonotic pathogens, as domestic cats could pass these infectious agents on to humans. Such cross-species transmission events from bats to domestic animals are well documented [9], [52].

### Virological investigations

For all tested human-pathogenic zoonotic viruses no infected bat could be detected in this study except lyssaviruses. Bat rabies is the only bat transmitted zoonosis in Europe that is known to have resulted in human cases [53]. Unlike in other mammals where lyssaviruses ultimately cause lethal rabies, in bats nonlethal lyssavirus infections may also lead to the development of immunity [47]. With the detection of EBLV-1 we confirm that this lyssavirus circulates among *E. serotinus* as previous studies showed [18]. In Germany, bat rabies is also caused by EBLV-2 and Bokeloh bat lyssavirus (BBLV) [54], [55], but while the latter was recently isolated from *M. nattereri*, EBLV-2 is associated with *M. daubentonii* and *M. dasycneme* [56]. The apparent absence of EBLV-2 and BBLV in the sampled bats is likely due to the fact that lyssavirus infections have a very low incidence in bat populations [18] and that the sample size was too limited, especially concerning the relevant species.

There is a high prevalence for herpesviruses in different insectivorous bat species in Germany (this study, [24]). Most of the previously described bat herpesviruses have been detected in low numbers in more than one bat species [24]. Here, we observed a high species-specific prevalence among herpesvirus-infected bats, indicating that a certain type of European bat herpesvirus is primarily associated with a single bat species. This is supported by BatGHV6 and BatGHV7 that were again only identified in their initial hosts *P. pipistrellus* and *P. auritus* (both sedentary), respectively, underlining the typical strong species-specificity of mammalian herpesviruses. However, species' range overlap and close inter-species contacts in bat roosts may result in cross-species transmission and could explain the observed overcoming of the species barrier (this study BatGHV5, [24]). Interspecies transmission have also been discussed for other mammalian herpesviruses, i.e. bovine and equine herpesviruses (e.g. [57], [58]). Furthermore, for RNA viruses (i.e. rabies virus) phylogenetic distance between different host species and overlap in geographic range have recently been demonstrated as strong predictors of host shifts and cross-species transmission in bats [59]. Some of the bat species (i.e. *N. noctula*, *P. pipistrellus*, and *P. nathusii*) in this study appear to be more susceptible to herpesvirus infection. In *N. noctula*, 3 different gammaherpesviruses (BatGHV3, 4, 5) with significant prevalence differences were recognized. Such type-specific differences in prevalence between the phylogenetically distant viruses BatGHV3 (13.0%) and BatGHV4 (33.8%) within one bat species indicates co-evolutionary virus-regulated mechanisms.

### Differences in parasite prevalence

Parasite infestation in wildlife often occurs without clinical effects, but severe infection can reduce host fitness either in terms of survival or reproductive success [60]. Most data on infection dynamics in bats came from parasite studies focusing on individual and seasonal variations in ectoparasite prevalence (e.g. [43]–[45], [61]). Host density, roost preference and movement pattern seem to be important factors explaining individual and species-specific parasite infestation rates in bats [43]–[45]. In European vespertilionid species, female-biased parasite loads are most likely associated with host physiology and differences in roosting behavior [42], [44]. We also found species-specific seasonal variations in ectoparasitic infestation, with *N. noctula* and *M. daubentonii* showing higher ectoparasite prevalence in spring and autumn compared to the breeding season (data not shown), which is in accordance with Zahn and Rupp [43].

Additional findings of our parasite analyses are distinct variations in ecto- and endoparasite prevalence in relation to bat species. Bats primarily roosting in trees or nest boxes were more frequently infested with ectoparasites like *N. noctula* (43%) and *M. daubentonii* (25%) compared to other species (range 5–12%) investigated in this study. High ectoparasite loads have generally been described in bats preferring enclosed roosts like burrows and cavities [61], [62], suggesting that structural characteristics and the microclimate of roosting habitats influence ectoparasite survival and re-infection of bat hosts. This assumption is in accordance with results of Pearce and O'Shea [63] who found differences in ectoparasite prevalence and intensity in *Eptesicus fuscus* in relation to environmental factors (i.e. temperature and humidity) of different roost sites. In contrast to these results, the endoparasite prevalence in European vespertilionid bats seems to be correlated with the body size of the bat species [26]. Species-specific variations in diet and prey selection could possibly effect endoparasite prevalence in insectivorous bats [64], as larger bats feed on insects of a wider size range including hard-bodied prey [65], [66]. This assumption is supported by the clear prevalence increase in subadult and adult bats compared to low endoparasite infection rates in juveniles primarily feeding on milk.

### Conclusion

A multitude of publications is restricted to pathogen presence or absence in different chiropteran species; here we demonstrate the impact of diseases and infectious agents on bats themselves. Alongside to trauma-associated mortality and undefined mortality cases, disease aspects represented one third of mortality causes in 486 investigated bats of 19 European vespertilionid species. By comparing pathology and bacteriology results, we were able to detect 22 different bacterial species that were clearly associated with disease in bats. At least 12% of all bats had died due to bacterial, viral and parasitic infections. Finally, we found clear seasonal and individual variations in disease prevalence and infection rates, indicating an increased susceptibility to infectious agents in female bats and juveniles during the maternity season. Our data emphasize and provide the basis for disease related studies in bat species on population level to elucidate the complexity of the ecology of infectious agents and host species likewise.

## Supporting Information

Table S1 **Bacteria isolated from bats found in Germany.**
(DOC)
