# Supplementary material for: Diseases and Causes of Death in European Bats: Dynamics in Disease Susceptibility and Infection Rates
Source: PLoS One. 2011 Dec 28;6(12):e29773. doi: 10.1371/journal.pone.0029773 (PMC3247292; doi:10.1371/journal.pone.0029773)
Supplement: Table S1 — Bacteria isolated from bats found in Germany. (DOC) [file pone.0029773.s001.doc]

**Table S1. Bacteria isolated from bats found in Germany**

| **Bacteria** | |  | **n** |  | **Bacteria** |  | **n** |
| --- | --- | --- | --- | --- | --- | --- | --- |
| **Family *Enterobacteriaceae*** | | |  |  | **Family *Pasteurellaceae*** | |  |
| *Hafnia* | *H. alvei* | | 48 |  | *Pasteurella* | *P. multocida* | 29 |
| *Serratia* | *S. liquefaciens* | | 43 |  |  | *P.* species B | 1 |
|  | *S. fonticola* | | 13 |  |  | *P. pneumotropica* | 1 |
|  | *S. marcescens* | | 10 |  | *Haemophilus*-like bacteria | | 4 |
|  | *S. plymuthica* | | 2 |  | **Other Gram-negative bacteria** | | |
|  | *S. proteamaculans* | | 1 |  | *Pseudomonas* | spp. ^a^ | 10 |
|  | sp.^a^ | | 1 |  | *Acinetobacter* | spp. ^a^ | 7 |
| *Klebsiella* | *K. oxytoca* | | 19 |  | *Aeromonas* | *A. hydrophila / caviae* | 6 |
|  | *K. mobilis* | | 6 |  | *Stenotrophomonas* | spp. ^a^ | 4 |
|  | *K. pneumoniae* | | 5 |  | *Vibrio* | spp. ^a^ | 2 |
| *Escherichia* | *E. coli* | | 15 |  | *Ralstonia* | sp. ^a^ | 1 |
|  | *E. vulneris* | | 1 |  | *Burkholderia* | sp. ^a^ | 1 |
|  | *E. blattae* | | 1 |  | *Myroides* | *M. odoratus* | 1 |
|  | spp. ^a^ | | 2 |  | **Family *Streptococcaceae*** | |  |
| *Enterobacter* | *E. cancerogenes* | | 11 |  | *Enterococcus* | *E. faecalis* | 63 |
|  | *E. amnigenus* | | 6 |  |  | *E. faecium* | 13 |
|  | *E. cloacae* | | 6 |  |  | *E. durans* | 1 |
|  | spp. ^a^ | | 3 |  |  | *E. avium* | 1 |
| *Pantoea* | spp. ^a^ | | 6 |  |  | *E. casseliflavus* | 1 |
| *Citrobacter* | *C. braakii* | | 2 |  |  | spp. ^a^ | 3 |
|  | *C. freundii* | | 1 |  | Alpha-haemolytic streptococci ^c^ | | 67 |
|  | *C. youngae* | | 1 |  | *Aerococcus* | *A. viridans* | 2 |
| *Rhanella* | *R. aquatilis* | | 3 |  | *Streptococcus* | *S. dysgalactiae* | 1 |
| *Salmonella* | *S.* Typhimurium ^b^ | | 2 |  | **Family *Micrococcaceae*** | |  |
|  | *S.* Enteritidis ^b^ | | 1 |  | Coagulase-negative staphylococci ^d^ | | 62 |
| *Morganella* | *M. morganii* | | 2 |  | *Staphylococcus* | *S. aureus* | 4 |
| *Cedecea* | *C. davisae* | | 2 |  |  | *S. intermedius* | 1 |
| *Moellerella* | *M. wisconsensis* | | 2 |  | **Family *Microbacteriaceae*** | |  |
| *Kluyvera* | spp. ^a^ | | 2 |  | *Microbacterium* | spp. ^a^ | 2 |
| *Yersinia* | *Y. pseudotuberculosis* | | 1 |  | *Leucobacter* | *L. aridicollis* | 1 |
|  | *Y. enterocolitica* | | 1 |  | **Other Gram-positive bacteria** | | |
| *Proteus* | *P. vulgaris* | | 1 |  | *Corynebacterium* | *C. afermentans* | 3 |
|  | sp. ^a^ | | 1 |  | *Propionibacterium* | *P. avidum* | 1 |
| *Erwinia* | sp. ^a^ | | 1 |  | *Clostridium* | *C. sordellii* | 1 |
| *Providencia* | *P. alcalifaciens* | | 1 |  | *Bacillus* | sp. ^a^ | 1 |

^a^ Bacterial isolates identified on the genus level.

^b^ *Salmonella enterica* serovar Typhimurium, *S. enterica* serovar Enteritidis.

^c^ *Lactococcus* spp., *Pediococcus* spp., *Leuconostoc* spp., *Carnobacterium* spp.

^d^ *Staphylococcus xylosus, S. sciuri, S. cohnii, S. haemolyticus, S. warneri, S. lentus, S. saprophyticus.*
